# Precise prediction of the sensitivity of platinum chemotherapy in SCLC: Establishing and verifying the feasibility of a CT-based radiomics nomogram

**DOI:** 10.3389/fonc.2023.1006172

**Published:** 2023-03-16

**Authors:** Yanping Su, Chenying Lu, Shenfei Zheng, Hao Zou, Lin Shen, Junchao Yu, Qiaoyou Weng, Zufei Wang, Minjiang Chen, Ran Zhang, Jiansong Ji, Meihao Wang

**Affiliations:** ^1^ Key Laboratory of Imaging Diagnosis and Minimally Invasive Intervention Research, Institute of Imaging Diagnosis and Minimally Invasive Intervention Research, The Fifth Affiliated Hospital of Wenzhou Medical University, Lishui, Zhejiang, China; ^2^ Department of Radiology, Key Laboratory of Intelligent Medical Imaging of Wenzhou, Institute of Aging, The First Affiliated Hospital of Wenzhou Medical University, Wenzhou Medical University, Wenzhou, Zhejiang, China; ^3^ Key Laboratory of Alzheimer’s Disease of Zhejiang, Wenzhou, Zhejiang, China; ^4^ Clinical College of The Affiliated Central Hospital, School of Medicine, Lishui University, Lishui, Zhejiang, China; ^5^ AI Research Department, Huiying Medical Technology Co., Ltd, Beijing, China

**Keywords:** radiomics, computed tomography, small cell lung cancer, chemotherapy, platinum

## Abstract

**Objectives:**

To develop and validate a CT-based radiomics nomogram that can provide individualized pretreatment prediction of the response to platinum treatment in small cell lung cancer (SCLC).

**Materials:**

A total of 134 SCLC patients who were treated with platinum as a first-line therapy were eligible for this study, including 51 patients with platinum resistance (PR) and 83 patients with platinum sensitivity (PS). The variance threshold, SelectKBest, and least absolute shrinkage and selection operator (LASSO) were applied for feature selection and model construction. The selected texture features were calculated to obtain the radiomics score (Rad-score), and the predictive nomogram model was composed of the Rad-score and the clinical features selected by multivariate analysis. Receiver operating characteristic (ROC) curves, calibration curves, and decision curves were used to assess the performance of the nomogram.

**Results:**

The Rad-score was calculated using 10 radiomic features, and the resulting radiomics signature demonstrated good discrimination in both the training set (area under the curve [AUC], 0.727; 95% confidence interval [CI], 0.627–0.809) and the validation set (AUC, 0.723; 95% CI, 0.562–0.799). To improve diagnostic effectiveness, the Rad-score created a novel prediction nomogram by combining CA125 and CA72-4. The radiomics nomogram showed good calibration and discrimination in the training set (AUC, 0.900; 95% CI, 0.844-0.947) and the validation set (AUC, 0.838; 95% CI, 0.534-0.735). The radiomics nomogram proved to be clinically beneficial based on decision curve analysis.

**Conclusion:**

We developed and validated a radiomics nomogram model for predicting the response to platinum in SCLC patients. The outcomes of this model can provide useful suggestions for the development of tailored and customized second-line chemotherapy regimens.

## 1 Introduction

Small cell lung cancer (SCLC), the most aggressive kind of lung cancer, accounts for approximately 14% of all lung cancer types and has a 5-year overall survival (OS) rate of just 6.7%. Due to its strong invasiveness, medication resistance, and the fact that no new, effective treatments have been developed in recent years (1, 2). Etoposide and platinum (EP) chemotherapy are the standard first-line therapies for SCLC, with initial response rates of 70–80% and high chemotherapeutic sensitivity. However, almost all patients will experience progression (3, 4). According to current studies, platinum-sensitive (PS) patients have a 15% to 20% better response rate to conventional second-line platinum chemotherapy than platinum-resistant (PR) patients, and their OS can be increased by 2-3 months (2, 5–7). For PS patients, the median PFS from the time of EP rechallenge as second-line treatment was 5.5 months, but PR patients had limited efficacy. Therefore, platinum reactivation is recommended for PS patients as second-line treatment, while PR patients are recommended to undergo topotecan treatment and other clinical trials. Thus, individualized second-line therapy based on an evaluation of platinum sensitivity is essential for improving the overall survival of SCLC patients (8–12).

Several studies have sought to use serum indicators and genetic tissue features to predict the responses to platinum in SCLC. SCLC is composed of four distinct subtypes, each of which reacts differently to platinum-based chemotherapy. The percentage of each subtype in the tumor influences how sensitive it is to platinum-based chemotherapy as a whole. However, the majority of SCLC tissue test samples are collected using needle biopsy, which unavoidably results in test variance and instability of prediction results (13). Other studies have attempted to use peripheral blood indices such as LDH and the systemic immune-inflammation index to predict the OS and PFS of SCLC (14, 15). However, the basic peripheral blood information is unconvincing, and these studies do not account for the tumor’s size, shape, location, or other relevant factors. Compared with the above methods, radiomics nomograms can be combined with radiomic and clinical features for noninvasive diagnosis, prognosis evaluation, and treatment response prediction. Previous studies have demonstrated that features based on radiomics are inextricably linked to underlying genomic patterns across a range of cancer types (16–18). Several studies using radiomics to predict platinum resistance in non-small cell lung cancer have been reported, and their radiomics models have shown excellent diagnostic efficacy (19–23). Nonetheless, there is no radiomics model for predicting platinum resistance in SCLC.

In this study, we aimed to develop and validate a CT-based radiomics nomogram that can provide individualized pretreatment prediction of the response to platinum treatment in SCLC, while effectively integrating image texture features and clinical factors. Using this nomogram, clinicians can enhance the treatment plan before initiating platinum-based chemotherapy and direct second-line therapy, optimize existing treatment combinations, and increase patient survival.

## 2 Materials and methods

### 2.1 Patients

**Figure 1:**
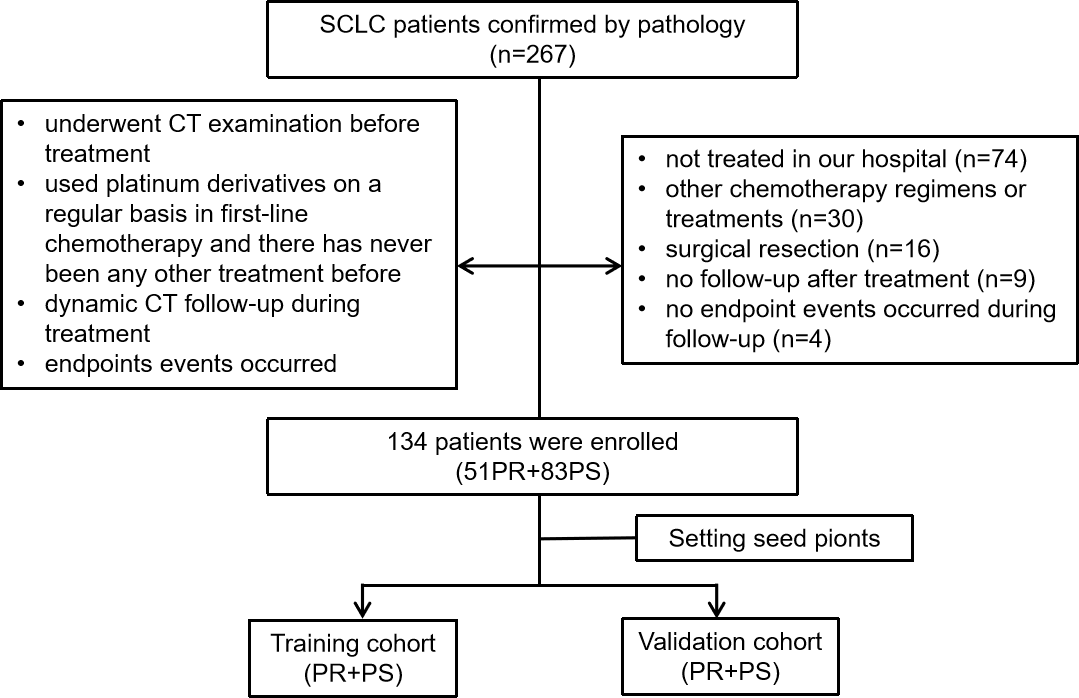
Flowchart of study enrollment.

The study was approved by the Institutional Review Board and Human Ethics Committee of the Fifth Affiliated Hospital of Wenzhou Medical University, and the requirement for informed consent was waived. Patients who were diagnosed with pathologically confirmed SCLC from February 2014 to November 2021 were enrolled. A total of 134 patients were included according to the following inclusion criteria: (1) they underwent a CT examination before treatment; (2) they used platinum derivatives on a regular basis in first-line chemotherapy and had never received any other treatment before; (3) dynamic CT follow-up was performed during treatment; and (4) endpoint events occurred. A total of 133 patients were excluded due to the following factors: (1) they were not treated in our hospital (n = 74); (2) they underwent other chemotherapy regimens or treatments (n = 30); (3) they underwent surgical resection (n = 16); (4) there was no follow-up after treatment (n = 9); and (5) no endpoint events occurred during follow-up (n = 4). Finally, 134 patients were selected for the present study. The flow of the case identification process is shown in **Figure 1**.

### 2.2 Endpoints

**Figure 2:**
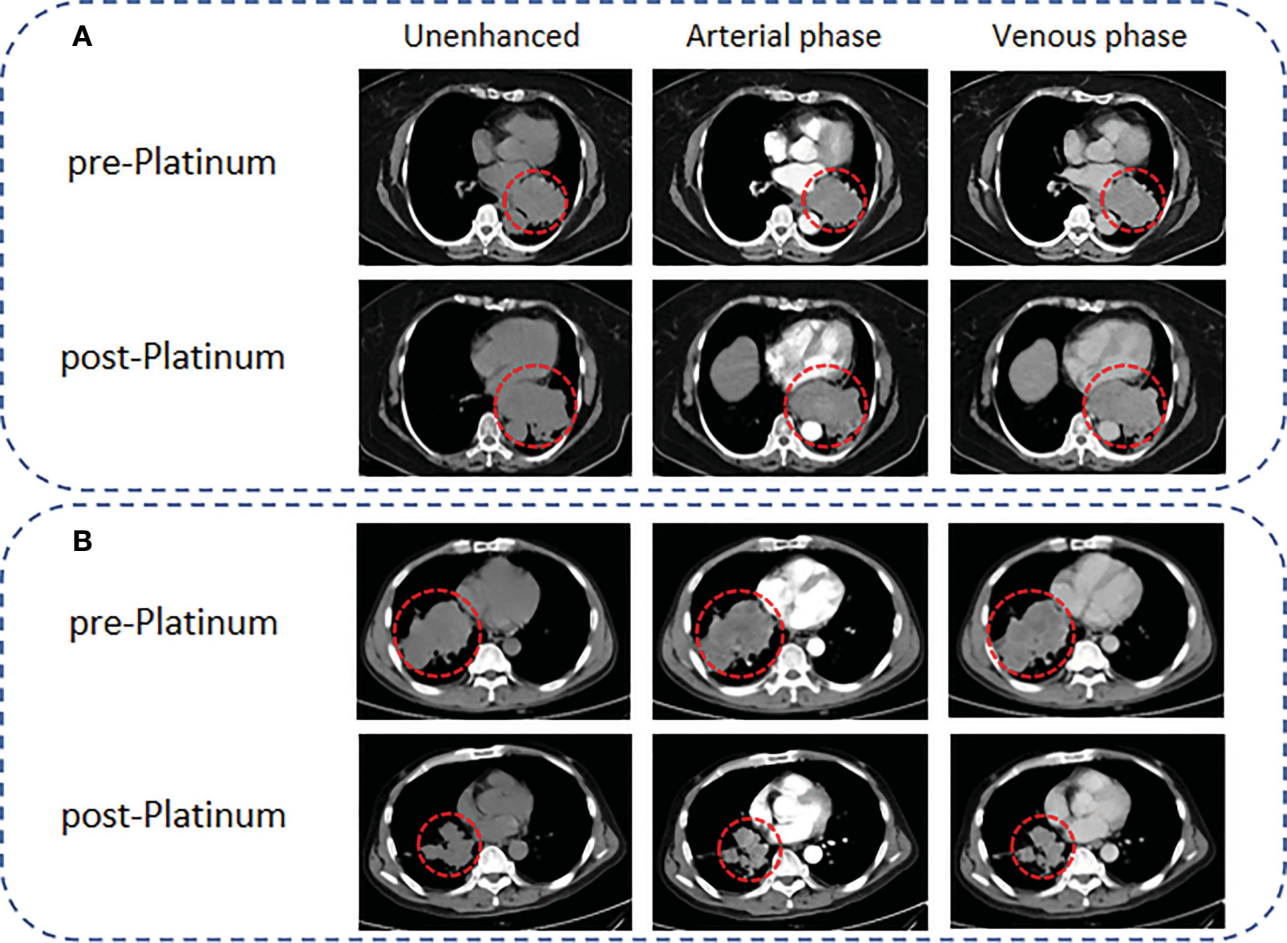
Representative CT images for PR and PS of SCLC patients according to the mRECIST criteria. **(A)** A 65-year-old female SCLC patient with a lesion diameter of 71 mm underwent CT scanning 1 week before EP chemotherapy, followed by CT scanning 2 months later. The lesion diameter increased to 97 mm, and the results showed that the patient presented with PR. **(B)** A 54-year-old male SCLC patient with a lesion diameter of 95 mm. CT scanning was performed 1 week before EP chemotherapy, and follow-up CT examinations were performed regularly after EP chemotherapy. Ten months later, the lesion diameter decreased to 56 mm, and the results showed that the patient presented with PS.

We evaluated the tumor response of SCLC patients who received CT examinations during platinum chemotherapy based on the modified Response Evaluation Criteria in Solid Tumors (mRECIST). The corresponding mRECIST responses were as follows: (1) complete response (CR): complete tumor disappearance; (2) partial response (PR): a minimum of 30% decrease in the sum of target lesion diameters; (3) progressive disease (PD): a minimum of 20% increase in the sum of target lesion diameters; and (4) stable disease (SD): neither PR nor PD. In this study, all patients underwent CT before and after platinum treatment, and the endpoint event was defined as the occurrence of PD. The patients were divided into PR and PS groups according to whether the time from platinum chemotherapy to the first PD was within 6 months. Representative CT images for PR and PS patients are shown in **Figure 2**.

### 2.3 CT image acquisition and interpretation

The patients underwent nonenhanced CT scans with a 256-slice Philips Brilliance iCT system prior to treatment (Philips Medical Systems). The following are the detailed acquisition parameter settings: tube voltage 120 kV, reference tube current 113 mAs, automatic millisecond technology, scanning field of view (SFOV) 15-20 cm, tube rotation time 0.75 s/circle, collimation width 80 mm (128×0.625 mm), reconstruction thickness 0.9 mm, reconstruction interval 0.45 mm, reconstruction matrix 1024× 1024, using the iDose3 iterative reconstruction algorithm.

Two thoracic radiologists with 5 and 15 years of experience (Y.S. and C.L.) independently conducted retrospective reviews. Disagreements were settled by a third radiologist who had 25 years of experience (J.J.). The image features included the following: (1) number of lesions and (2) volume, measured using the Extended Brilliance Workspace and Lung Nodule Assessment software (Philips); (3) location: central or peripheral; (4) morphology: regular or irregular; (5) shape: regular or irregular; (6) lobulation (present/absent); (7) necrosis (present/absent); (8) hydrothorax (present/absent); (9) intratumoral calcification (present/absent); (10) staging (limited-stage/extensive-stage); and (11) metastasis (lymph den/bone/parenchyma organ/cardiovascular/pleural and pericardium).

### 2.4 Tumor segmentation of volumes of interest and extraction of radiomic features

**Figure 3:**
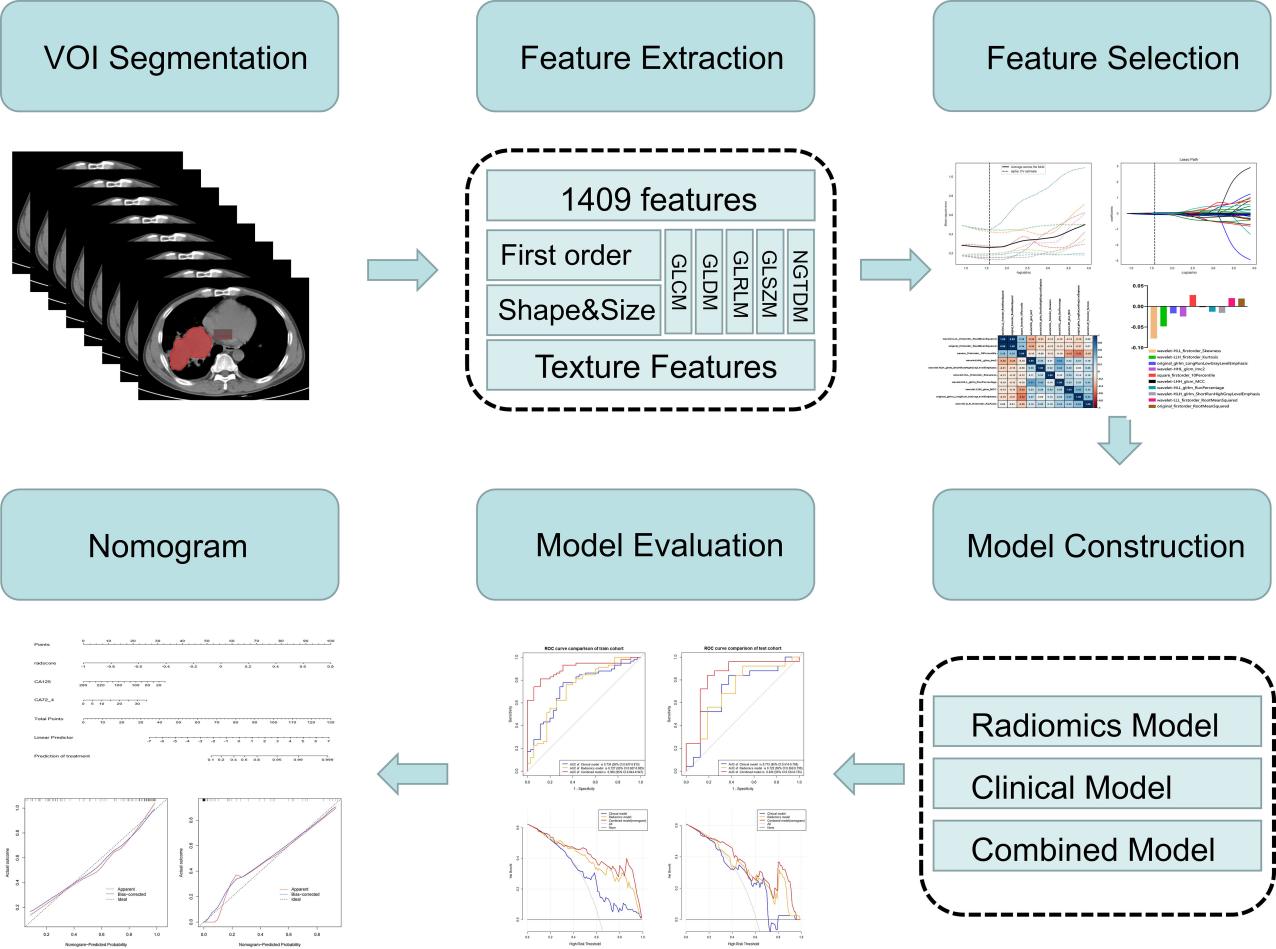
Flowchart of the study.

The radiomics workflow is shown in **Figure 3**. Tumors and mediastinal lymph nodes fused with tumors in the mediastinal window were included in the volume of interest (VOI). First, a radiologist (reader 1, Y. S, a radiologist with five years of chest imaging experience) manually annotated 3D tumor VOIs around the largest lesion using the Radcloud platform (Huiying Medical Technology Co., Ltd, http://mics.radcloud.cn). To evaluate the reproducibility of the extracted features, reader 2 (C. L, a radiologist with 15 years of chest imaging experience) independently segmented 10% of lesions randomly selected from both the PR and PS groups.

For each VOI on our CT images, 1,409 radiomic features were extracted using a tool from the Radcloud platform, which extracted radiomic features from medical image data with a large panel of engineered hard-coded feature algorithms (https://pyradiomics.readthedocs.io/en/latest/features.html). The 1,409 features obtained were divided into four main categories: first-order statistics, shape, texture [gray-level cooccurrence (GLCM), gray-level run length (GLRLM), gray-level size zone (GLSZM), neighboring gray tone difference (NGTDM), gray-level dependence (GLDM), Matrices], and higher-order statistics (Laplacian of Gaussian, wavelet, square, square root, logarithm, exponential, gradient, and local binary pattern filters) features.

The intraclass correlation coefficient (ICC) was used to validate the reproducibility of extracted features from the two radiologists. Radiomic features with intra-ICCs >0.75 were selected for the subsequent statistical analysis.

### 2.5 Construction of a radiomics signature and assessment of performance

In the imaging and storage of medical images, to make the intensity information consistent, the following formula was used to normalize all the radiomic features of CT images.


$$f\left(x\right)=\frac{s\left(x-u_{x}\right)}{\sigma_{x}}$$

Where *f*(*x*) is the normalized intensity, *x* is the original intensity, *µ* and *σ* are the mean value and variance, respectively, and *s* represents an optional scaling whose default is 1.

The samples were randomly divided into a training cohort (n=58, 70%) and a validation cohort (n=25, 30%). To reduce the redundant features, the feature selection methods included the variance threshold, SelectKBest, and the least absolute shrinkage and selection operator (LASSO). For the variance threshold method, the threshold is 0.8, so that the eigenvalues of the variance smaller than 0.8 are removed. The SelectKBest method, which is a single-variable feature selection method, uses the p value to analyze the relationship between the features and the classification results; all the features with a p value smaller than 0.05 are used. For the LASSO model, L1 regularization is used as the cost function, the error value of cross-validation is 10, and the maximum number of iterations is 1,000. Subsequently, the radiomic parameters with nonzero coefficients in the LASSO model generated by the entire training cohort with the optimal a were selected. The radiomics signature (i.e., Rad-score) was computed for each lesion by a linear combination of the selected features as weighted by their respective quotient.

### 2.6 Construction and internal validation of the nomogram model

The variables, including clinical factors, conventional CT findings, and Rad-scores between the samples of platinum-resistant groups and platinum-sensitive groups with significant differences, were analyzed *via* multivariate logistic regression to build the radiomics nomogram. The performance of the nomogram was evaluated by plotting receiver operating characteristic curves.

The Hosmer−Lemeshow test was used to evaluate the goodness-of-fit of the nomogram. The classification accuracy between the predicted probability and the observed results was evaluated using calibration curves. The diagnostic performance of the nomogram was assessed by evaluating the AUC, sensitivity, specificity, and accuracy. The AUC between the optimized signature and the nomogram was evaluated by using the DeLong test. Decision curve analysis (DCA) was performed to evaluate the clinical utility of the nomogram.

### 2.7 Statistical analysis

All quantitative features were analyzed with SPSS 25. P<0.05 was considered as statistically significant.

Categorical variables are shown as frequencies, and continuous variables are presented as the mean and standard deviation or median and interquartile range. The χ2 test was used to analyze the categorical variables, the t test was applied to analyze the continuous variables with a normal distribution, and the Mann−Whitney U test was used for variables with an abnormal or unknown distribution. Multivariable logistic regression analysis was used to select the independent prognostic factors. The performance of the model was assessed in the primary and validation cohorts. The discrimination of the signature was measured by the area under the curve (AUC).

The ICC was graded as follows: poor (<0.20), moderate (0.20–0.40), fair (0.40–0.60), good (0.60–0.80), or very good (0.80–1.00).

Statistical analyses were performed using SPSS software (Ver. 25, IBM, Armonk, New York), SigmaPlot (Ver. 14.0), R software package (Ver. 3.5.2, R Development Core Team: https://www.r-project.org/), and the Python scikit-learn package (Ver. 3.7, scikit-learn Ver. 0.21, http://scikit-learn.org/).

## 3 Results

### 3.1 Clinical factors of the patients and construction of the clinical factor model

The baseline clinical characteristics of the patients are summarized in **Table 1**. A total of 134 patients were enrolled in this study: 51 patients with PR and 83 patients with PS. The mean ages were 62.71 ± 9.38 and 61.28 ± 7.33, respectively. Univariate analysis showed that NSE, CEA, CA125, CA72-4, CA199, and TG were significantly different between the two groups. Subsequently, multivariate analysis suggested that CA125 (OR: 0.98, 95% CI: 0.977-0.998, P =0.022) and CA72-4 (OR: 1.172, 95% CI: 1.023-1.341, P =0.022) were independent predictors of SCLC with PS (**Table 2**). The ROC curves of CA125, CA72-4 and the clinical model are shown in **Figure S1**.

**Table 1:** Baseline characteristics of the patients in the PR and PS groups.

Variables	PR (51)	PS (83)	t/χ^2^/U	*P*
Sex			0.063	0.802
Male	45 (88%)	72 (87%)		
Female	6 (12%)	11 (13%)		
Age	62.71 ± 9.38	61.28 ± 7.33	0.983	0.327
BMI/kg·m-2	22.06 (20.20, 25.00)	22.53 (20.57, 24.61)	0.472	0.637
Smoking	38 (75%)	62 (75%)	<0.001	0.981
Superior vena cava syndrome	3 (6%)	5 (6%)	<0.001	1.000
Spinal cord compression	3 (6%)	5 (6%)	<0.001	1.000
Ki67	80% (70%, 85%)	80% (70%, 85%)	0.576	0.565
Tumor number			0.737	0.692
1	41 (80%)	69 (83%)		
2	1 (2%)	3 (4%)		
≥3	9 (18%)	11 (13%)		
Tumor volume	115.06 (14.50, 247.30)	65.99 (18.72, 160.40)	1.191	0.233
Intratumoral calcification	3 (6%)	2 (2%)	0.314	0.575
Tumor location			<0.001	0.987
Central	40 (78%)	65 (78%)		
Peripheral	11 (22%)	18 (22%)		
Tumor morphology			1.615	0.532
Regular	16 (31%)	25 (30%)		
Irregular	35 (69%)	58 (70%)		
Lobulated			<0.001	1.000
Absent	2 (4%)	4 (5%)		
Present	49 (96%)	79 (95%)		
Necrosis			0.285	0.594
Absent	27 (53%)	41 (48%)		
Present	24 (47%)	43 (52%)		
Hydrothorax			1.941	0.164
Absent	27 (53%)	55 (65%)		
Present	24 (47%)	29 (35%)		
Staging			0.202	0.653
LS	25 (49%)	45 (53%)		
ES	26 (51%)	39 (47%)		
Metastasis				
Lymph den	49 (96%)	73 (88%)	1.659	0.198
Bone	9 (18%)	6 (7%)	3.449	0.063
Parenchyma organ	12 (24%)	14 (17%)	0.897	0.344
Cardiovascular	12 (24%)	16 (19%)	0.346	0.557
Pleural and pericardium	7 (14%)	8 (10%)	0.531	0.466
NSE	37.30 (22.20, 103.60)	31.80 (18.20, 55.90)	1.991	**0.046**
CEA	7.30 (2.90, 18.60)	3.80 (2.20, 6.50)	2.390	**0.017**
Pro-GRP	714.9 (129.90, 3212.20)	597.80 (156.90, 1953.10)	0.660	0.509
CYFRA-211	3.20 (2.30, 5.20)	2.90 (2.00, 4.40)	1.407	0.159
CA125	33.40 (18.20, 66.60)	17.30 (13.10, 31.10)	4.443	**<0.001**
CA72-4	1.60 (1.00, 2.60)	2.50 (1.20, 5.60)	2.726	**0.006**
CA199	23.40 (5.60, 48.80)	12.10 (4.80, 23.20)	2.809	**0.005**
FER	249.70 (156.00, 491.80)	280.50 (188.30, 387.90)	0.332	0.740
SCC	0.90 (0.50, 1.10)	0.70 (0.60, 1.00)	0.600	0.549
ApoB/ApoA	0.76 (0.63, 0.82)	0.67 (0.60, 0.80)	1.241	0.215
HDL	1.12 (0.90, 1.32)	1.12 (0.97, 1.27)	0.133	0.894
LDL	2.47 (2.12, 3.10)	2.37 (2.12, 3.10)	0.500	0.617
TG	0.98 (0.77, 1.43)	1.40 (1.12, 2.01)	4.015	**<0.001**

NSE, neuron-specific enolase; CEA, carcinoembryonic antigen; pro-GRP, progastrin-releasing peptide; CA125, carbohydrate antigen 125; CA72-4, carbohydrate antigen 72-4; CA199, carbohydrate antigen 199; FER, ferroprotein; SCC, squamous cell carcinoma; ApoB, apolipoprotein B; ApoA, apolipoprotein A; HDL, high-density lipoprotein; LDL, low-density lipoprotein; TG, triglyceride.

**Table 2:** Univariate and multivariate analyses of clinical factors.

Characteristic	Univariate			Multivariate		
	OR	95% CI	*P*	OR	95% CI	*P*
NSE	0.994	0.988-0.999	0.024	0.994	0.987-1.000	0.063
CEA	0.982	0.962-1.002	0.078			
CA125	0.986	0.975-0.997	0.014	0.987	0.977-0.998	0.022
CA72-4	1.187	1.037-1.359	0.013	1.172	1.023-1.341	0.022
CA199	0.995	0.987-1.003	0.212			
TG	1.043	0.913-1.190	0.538			

### 3.2 Feature extraction, selection, and radiomic signature building

**Figure 4:**
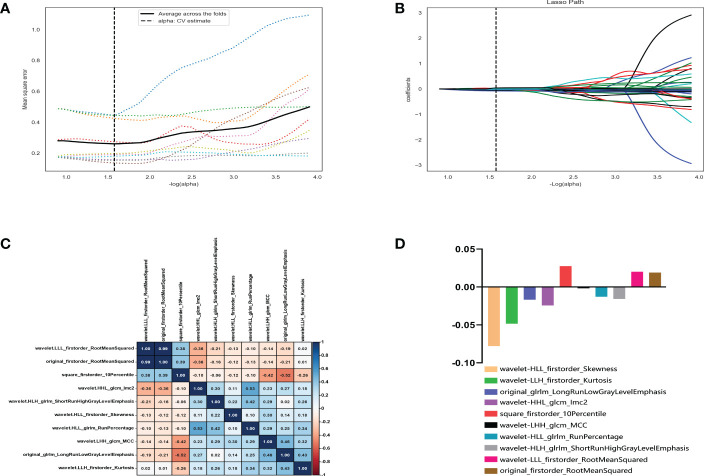
Radiomic feature selection using the variance threshold, SelectKBest and selection operator (LASSO) regression model. LASSO regression model on CT images. The mean square error on each fold in the tenfold cross-validation method and the optimal value of the lasso tuning parameter (-log (α)=1.574,α= 2.978) were found **(A)**. The vertical line was plotted with 10 selected radiomic features **(B)**. The 10 radiomic features were selected after dimension reduction **(C, D)**.

**Figure 5:**
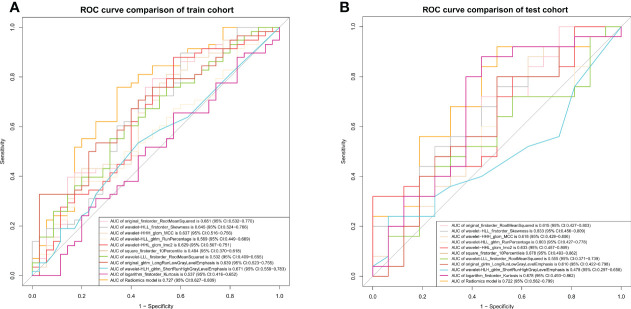
ROC curves of the radiomics model. The ROC curves of the radiomics model in the training **(A)** and validation **(B)** cohorts.

Of the 1409 radiomic features extracted from CT images, 1186 were demonstrated to have good interobserver agreement, with intra-ICCs >0.75. A total of 1107 radiomic features by variance threshold were enrolled in SelectKBest to select the most valuable 60 features. Finally, 10 features were screened out by LASSO to build the radiomic signature model. The optimal parameter λ of each fold and the selected features of the corresponding fold are shown in **Figure 4**. The ROC curves of the 10 radiomic features and radiomics model are shown in **Figure 5**.

Based on these 10 features and their regression coefficients, the radiomics score (Rad-score) formula was constructed as follows: Rad-score = feature * coefficient (**Table 3**).

**Table 3:** Description of the selected radiomic features with their associated feature group and filter.

Radiomic feature	Radiomic class	Filter	Coefficient
Skewness	firstorder	wavelet-HLL	-0.0778821372989
Kurtosis	firstorder	wavelet-LLH	-0.048467472885
LongRunLowGrayLevelEmphasis	glrlm	original	-0.0168388737795
Imc2	glcm	wavelet-HHL	-0.0243113156389
10Percentile	firstorder	square	0.0276043499311
MCC	glcm	wavelet-LHH	-0.00170586466044
RunPercentage	glrlm	wavelet-HLL	-0.0129710574977
ShortRunHighGrayLevelEmphasis	glrlm	wavelet-HLH	-0.0156495283052
RootMeanSquared	firstorder	wavelet-LLL	0.0201114297855
RootMeanSquared	firstorder	original	0.0190099678839

Imc2, Informational Measure of Correlation 2; MCC, Maximal Correlation Coefficient; glrlm, gray level tun length matrix; glcm, gray-level cooccurrence matrix.

### 3.3 Radiomics nomogram building and assessment of the performance of different models

**Figure 6:**
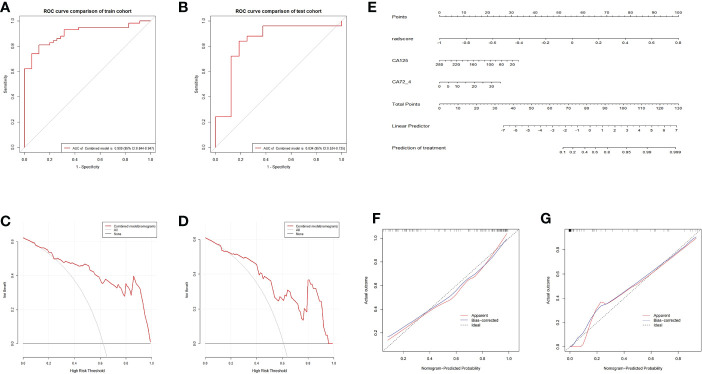
ROC and curve decision curve analysis of the nomogram model. The radiomics nomogram and calibration curves for the radiomics nomogram. The ROC curves of the nomogram model in the training **(A)** and validation **(B)** sets and the decision curve analysis for the nomogram model in the training **(C)** and validation **(D)** sets. The radiomics nomogram, combining CA125, CA72-4, and Rad-score, was developed in the training cohort **(E)**. Calibration curves for the radiomics nomogram in the training **(F)** and validation **(G)** cohorts. Calibration curves indicate the goodness-of-fit of the nomogram. The 45° gray line represents the ideal prediction, and the blue lines and red lines represent the performance of the corrected and apparent bias, respectively. The closer the line approaches the ideal prediction line, the better the predictive efficacy of the nomogram.

The ROC and decision curves of the nomogram model are shown in **Figures 6A–D**. The CA125, CA72-4, and Rad-score were incorporated into the construction of the radiomics nomogram (**Figure 6E**). **Figures 6F, G** shows the calibration curve of the nomogram. The Nomo-scores for each patient are shown in **Figure S2**. The AUC of the clinical model was higher than that of the radiomics model in the training cohort, whereas the AUC value of the radiomics model was higher than that of the clinical model in the validation cohort. The AUC value of the nomogram model was significantly higher than that of the clinical and radiomics models in the training cohort and verification cohort. The calibration curve showed good calibration in the training cohort and validation cohort (**Figure S3**). The radiomics nomogram showed the highest net benefit of the three models.

The radiomics signatures based on the nomogram model showed high performance in differentiating between platinum-resistant groups and platinum-sensitive groups, with an AUC of 0.900 (95% CI, 0.844-0.947; sensitivity, 83.61%; specificity, 78.13%; accuracy, 81.72%) in the training cohort and 0.838 (95% CI, 0.534-0.735; sensitivity, 68.57%; specificity, 83.33%; accuracy, 70.73%) in the validation cohort. The AUC of the CT image model was 0.727 (95% CI, 0.627-0.809; sensitivity, 73.85%; specificity, 64.29%; accuracy, 70.97%) in the training cohort and 0.723 (95% CI, 0.562-0.799; sensitivity, 71.88%; specificity, 77.78%; accuracy, 73.17%) in the validation dataset. The AUC of the clinical model was 0.734 (95% CI, 0.637–0.814; sensitivity, 65.82%; specificity, 57.14%; accuracy, 64.52%) in the training cohort and 0.715 (95% CI, 0.514–0.756; sensitivity, 70.00%; specificity, 63.64%; accuracy, 68.29%) in the test dataset. For the combined radiomics signature, the Hosmer−Lemeshow test yielded P values of 0.219 and 0.308 in the training and validation cohorts, respectively, indicating no departure from a good fit.

## 4 Discussion

The standard first-line therapy for SCLC is platinum-based chemotherapy, which has a 70–80% success rate and often a very pronounced early effect (3, 4). However, the disease will progress quickly, on average, six months after the first treatment has been administered (2). Current management advice is that PR patients should try clinical trial medication such as topotecan or lurbinectedin because they would gain little from an EP regimen, whereas PS patients should be restimulated with an EP regimen (6). To increase the overall survival rate, it is crucial to evaluate the tumor’s response to platinum chemotherapy and choose a suitable second-line treatment prior to first-line therapy (24, 25). In this study, we established a CT-based, noninvasive radiomics nomogram model that incorporates the radiomics signature and clinical factors to predict a customized platinum response in SCLC patients. Overall, our study serves as an example of precision medicine and can influence treatment options.

In our study, mediastinal window texture characteristics in patients with SCLC were extracted using radiological methods, and 1409 potential radiological features were chosen for further investigation. We were able to greatly enhance the number of texture characteristics by utilizing 3D annotation, which allowed us to avoid missing any crucial aspects altogether. Wavelet-based characteristics have been proposed as a tool for illness diagnosis and predicting therapy response (26, 27). GLCM and GLRLM are both matrix-based features: GLCM describes the pairwise arrangement of voxels with the same gray value and is used to highlight local heterogeneity information; GLRLM is used to measure the distribution of high gray values, and the GrayLevelEmphasis value is expected to be larger for images with higher gray values. Our Rad-score includes two GLSZM features, MCC and GLCM. The MCC represents the complexity of the texture, and the lower the value, the more complex the texture. In this study, the MCC value of the sensitive group was lower, indicating that the lesion heterogeneity in the sensitive group was higher, and thus, the probability of a response to the treatment outcome was higher.

Meanwhile, the potential 1409 candidate radiomic features were finally reduced to 10 potential predictors by shrinking the regression coefficients with the LASSO method for further integration to form the Rad-score, which contains effective biological information and could reflect the heterogeneity of the tumor. The radiomics signature demonstrated good discrimination in both the training set (AUC, 0.727; 95% CI, 0.627-0.809) and the validation set (AUC, 0.723; 95% CI, 0.562-0.799). Several previous studies have demonstrated that the Rad-score can effectively predict the prognosis of patients due to its high correlation with tumor biological characteristics (28). Several radiomic model prediction algorithms have been developed in the past to predict tumor response to medications, including platinum-based chemotherapeutics, in a variety of cancers (16, 17, 19, 20). A recent study showed that the computed tomography-based radiomics signature was closely associated with the PFS of SCLC; however, this study primarily concentrated on PFS prediction and made no recommendations to enhance PFS, which only offered minimal clinical support (23). These preliminary studies have further confirmed that the texture feature-based radiomics method of SCLC is feasible for predicting platinum responsiveness. Additionally, our study expands on these findings to achieve more significant outcomes with regard to clinical requirements and increased patient survival.

To improve the prediction efficacy, predictors beyond radiomics should also be incorporated with the radiomics signature to further increase the power of the decision support model. In previous studies, patient prognosis was influenced by characteristics such as patient sex, smoking history, tumor stage, and other variables; however, in our study, these variables had no impact on the tumor’s sensitivity to platinum-based chemotherapy. NSE, Pro-GRP, and CYFRA 21-1 are all linked to the prognosis of SCLC; however, they also cannot predict platinum resistance. As a result, CA125 and CA72-4 with corresponding odds ratios of 0.987 and 1.172 were selected by multivariable logistic regression analysis. The ORs suggest that the higher the CA125 and CA72-4 levels are, the greater the probability of a favorable response to platinum treatment in SCLC. The clinical phases of SCLC were linked to CA125 (29). According to the literature, a higher level of CA125 can indicate a better impact of first-line treatment (30). Although Ca72-4 can predict the degree of differentiation in gastric cancer (31–33), no studies have found a link between it and small cell lung cancer. The baseline expression of CA 125 and CA72-4 in SCLC can predict platinum resistance, according to our findings.

After selecting candidate predictors using multivariate logistic regression analysis, a nomogram model was built that included radiomics signatures, CA125, and CA72-4. Of note, our radiomics nomogram showed favorable discrimination (AUC 0.900) in the training cohort, which was further validated in the internal validation cohorts (AUC 0.834). Furthermore, DCA showed a higher overall net benefit of the radiomics model, thus highlighting its value as a better tool for assisting in clinical decision-making. Using the radiomics nomogram model, if a patient is predicted to have a favorable response to platinum, second-line platinum chemotherapy should be recommended; if not, immune checkpoint inhibitors are a good alternative (11, 34, 35). This is particularly important for those with PR, since doctors can choose other treatment options at an earlier stage to prevent tumor progression due to drug resistance and improve recurrence-free survival. However, our study has several limitations. First, given the retrospective nature of this study, selection bias may exist. Second, the training/testing cohort is tiny. Due to morbidity, the sample size is smaller than other tumor type radiomics research samples but similar to those in SCLC radiomics studies. Larger datasets are needed to verify and improve our results, and external validation of our model’s performance with an independent cohort from other institutions is necessary.

In summary, we developed and validated a radiomics model that incorporates the pretreatment CT-based radiomics signature and clinical variables for the prediction of the response to platinum treatment in patients with SCLC. This study can assist patients in customizing second-line chemotherapy, improve clinical decision-making, and increase patient survival. Additionally, this research could be utilized to forecast second-line therapy responsiveness and support the development of third-line treatment approaches. It offers a wide range of potential applications and is also applicable to different tumor types.

## Data availability statement

The raw data supporting the conclusions of this article will be made available by the authors, without undue reservation.

## Ethics statement

The studies involving human participants were reviewed and approved by Institutional Review Board and Human Ethics Committee of Lishui Central Hospital. Written informed consent for participation was not required for this study in accordance with the national legislation and the institutional requirements.

## Author contributions

MW and JJ designed the study. SZ, HZ, LS and JY collected the data and drafted the paper. RZ, YS, QW, CL and ZW analyzed the data and made the figures. YS, MC, CL, JJ and MW revised the paper. All authors contributed to the article and approved the submitted version.
