# Supplementary material for: Precise prediction of the sensitivity of platinum chemotherapy in SCLC: Establishing and verifying the feasibility of a CT-based radiomics nomogram
Source: Front Oncol. 2023 Mar 16;13:1006172. doi: 10.3389/fonc.2023.1006172 (PMC10061075; doi:10.3389/fonc.2023.1006172)
Supplement: Supplementary file 1 [file DataSheet_1.docx]

Supplementary Material

# Supplementary Figures and Tables

## Supplementary Figures

**
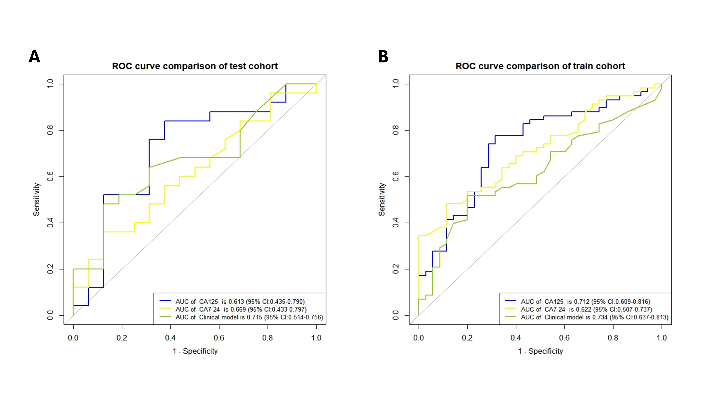
**

**Supplementary Figure 1.** ROC curves of the clinical model. The ROC curves of the clinical model in the training (A) and validation (B) sets.

**
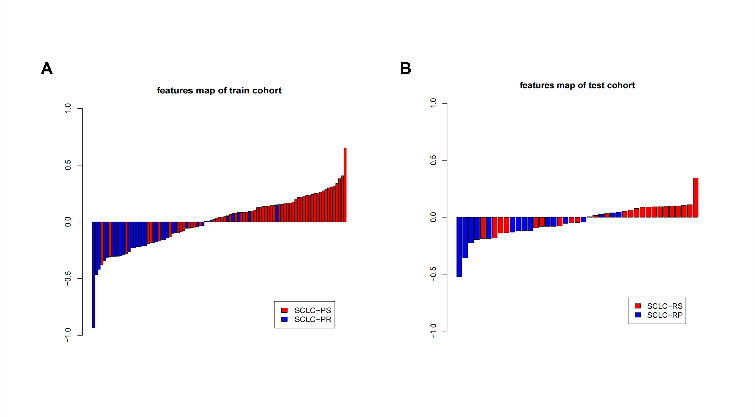
**

**Supplementary Figure 2.** The nomogram scores for each patient in the training (A) and validation (B) cohorts. The nomogram scores for each patient in the training and validation sets. Red bars represent the PS scores, while blue bars represent the PR scores.

**
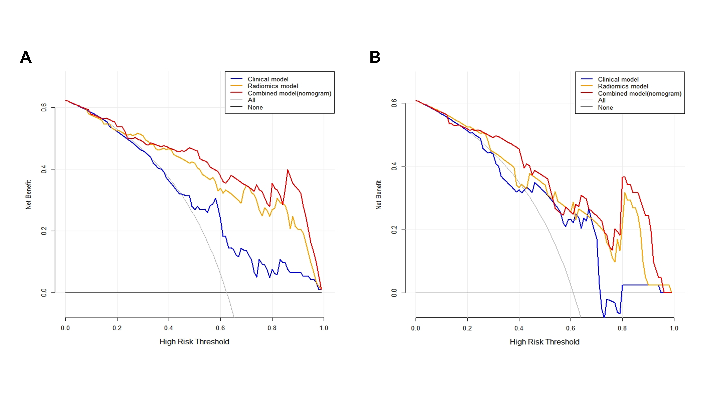
**

**Supplementary Figure 3.** ROC and curve decision curve analysis of the three models in the training (A) and validation (B) cohorts. The y-axis indicates the net benefit; the x-axis indicates threshold probability. The red line, blue line, and yellow line represent the net benefit of the radiomics nomogram, the clinical factor model, and the radiomics signature, respectively. The radiomics model had the highest net benefit compared with the other two models and simple diagnoses.

**Table S1** Baseline characteristics of the patients in the training and validation groups

| Variables | Training set（35/58） | Validation set（16/25） | t/Χ^2^/U | P |
| --- | --- | --- | --- | --- |
| Sex |  |  | 0.013 | 0.910 |
| Male | 81 (87%) | 36 (88%) |  |  |
| Female | 12 (13%) | 5 (12%) |  |  |
| Age | 61.32±8.00 | 62.95±8.52 | 1.039 | 0.302 |
| BMI /kg·m-2 | 22.45 (20.39, 24.94) | 22.19 (20.40, 24.55) | -0.519 | 0.604 |
| Smoking | 65 (70%) | 35 (85%) | 3.598 | 0.058 |
| Superior vena cava syndrome | 6 (7%) | 2 (5%) | <0.001 | 1.000 |
| Spinal cord compression | 7 (8%) | 1 (2%) | 0.562 | 0.453 |
| Ki67 | 80% (70%, 85%) | 80% (70%, 87.5%) | 0.576 | 0.565 |
| Tumor number |  |  | 3.002 | 0.223 |
| 1 | 75 (81%) | 35 (85%) |  |  |
| 2 | 4 (4%) | 0 (0%) |  |  |
| ≥3 | 14 (15%) | 6(15%) |  |  |
| Tumor volume | 115.06 (14.50, 247.30) | 65.99 (18.72, 160.40) | 1.191 | 0.233 |
| Intratumoral calcification | 3 (3%) | 2 (5%) | <0.001 | 1.000 |
| Tumor location |  |  | 4.589 | 0.11 |
| Central | 25 (27%) | 16 (39%) |  |  |
| Peripheral | 68 (73%) | 25 (61%) |  |  |
| Tumor morphology |  |  | 1.615 | 0.532 |
| regular | 16 (31%) | 25 (30%) |  |  |
| irregular | 35 (69%) | 58 (70%) |  |  |
| Lobulated |  |  | 2.276 | 0.131 |
| absent | 2 (2%) | 4 (10%) |  |  |
| present | 91 (98%) | 37 (90%) |  |  |
| Necrosis |  |  | 0.879 | 0.349 |
| absent | 44 (47%) | 23 (56%) |  |  |
| present | 49 (53%) | 18 (44%) |  |  |
| Hydrothorax |  |  | 0.468 | 0.494 |
| absent | 58 (62%) | 23 (56%) |  |  |
| present | 35 (38%) | 18 (44%) |  |  |
| Staging |  |  | 0.174 | 0.677 |
| LS | 49 (53%) | 20 (49%) |  |  |
| ES | 44 (47%) | 21 (51%) |  |  |
| Metastasis |  |  |  |  |
| Lymph den | 84 (90%) | 38 (93%) | 0.013 | 0.910 |
| bone | 11 (11.8%) | 4 (10%) | 0.003 | 0.958 |
| Parenchyma organ | 15 (16%) | 11 (27%) | 2.083 | 0.149 |
| Cardiovascular | 20 (22%) | 8 (20%) | 0.068 | 0.794 |
| Pleural and pericardium | 10(11%) | 5 (12%) | <0.001 | 1.000 |
| NSE | 33.60 (21.15, 66.80) | 39.50 (20.85, 73.750) | -0.628 | 0.530 |
| CEA | 4.50 (2.60, 12.35) | 4.00 (2.10, 14.60) | -0.118 | 0.906 |
| Pro-GRP | 597.80 (105.25, 2135.05) | 714.90 (280.15, 2679.50) | -0.935 | 0.350 |
| CYFRA-211 | 2.70 (2.00, 4.65) | 3.40 (2.25, 4.70) | -1.137 | 0.225 |
| CA125 | 21.10 (13.70, 34.35) | 20.50 (14.70, 67.90) | -0.862 | 0.389 |
| CA724 | 1.90 (1.20, 5.00) | 2.10 (1.15, 3.45) | -0.457 | 0.648 |
| CA199 | 15.50 (5.05, 32.25) | 14.50 (5.10, 32.55) | -0.138 | 0.891 |
| FER | 264.50 (171.55, 390.20) | 290.40 (215.70, 410.85) | -1.282 | 0.200 |
| SCC | 0.80 (0.60, 1.00) | 0.70 (0.60, 1.00) | -0.365 | 0.715 |
| ApoB/ApoA | 0.70 (0.60, 0.80) | 0.67 (0.63, 0.84) | -0.454 | 0.650 |
| HDL | 1.12 (0.98, 1.26) | 1.16 (0.87, 1.32) | -0.082 | 0.935 |
| LDL | 2.46 (2.17, 3.13) | 2.30 (2.01, 2.91) | -1.229 | 0.219 |
| TG | 1.33 (0.90, 1.63) | 1.13 (0.93, 1.51) | -0.768 | 0.443 |

NSE, neuron-specific enolase; CEA, carcinoembryonic antigen; pro-GRP, progastrin-releasing peptide; CA125, carbohydrate antigen 125; CA72-4, carbohydrate antigen 72-4; CA199, carbohydrate antigen 199; FER, ferroprotein; SCC, squamous cell carcinoma; ApoB, apolipoprotein B; ApoA, apolipoprotein A; HDL, high-density lipoprotein; LDL, low-density lipoprotein; TG, Triglyceride.
